# A Critical Review of Remote Sensing Approaches and Deep Learning Techniques in Archaeology

**DOI:** 10.3390/s23062918

**Published:** 2023-03-08

**Authors:** Israa Kadhim, Fanar M. Abed

**Affiliations:** 1 Environment and Sustainability Institute, University of Exeter, Penryn Campus, Penryn, Cornwall TR10 9FE, UK; 2 College of Engineering, University of Baghdad, Baghdad 10001, Iraq

**Keywords:** digital archaeology, Remote Sensing, hidden features, digital preservation, standalone approaches, fusion approaches, photogrammetry, LiDAR, Deep Learning

## Abstract

To date, comprehensive reviews and discussions of the strengths and limitations of Remote Sensing (RS) standalone and combination approaches, and Deep Learning (DL)-based RS datasets in archaeology have been limited. The objective of this paper is, therefore, to review and critically discuss existing studies that have applied these advanced approaches in archaeology, with a specific focus on digital preservation and object detection. RS standalone approaches including range-based and image-based modelling (e.g., laser scanning and SfM photogrammetry) have several disadvantages in terms of spatial resolution, penetrations, textures, colours, and accuracy. These limitations have led some archaeological studies to fuse/integrate multiple RS datasets to overcome limitations and produce comparatively detailed outcomes. However, there are still knowledge gaps in examining the effectiveness of these RS approaches in enhancing the detection of archaeological remains/areas. Thus, this review paper is likely to deliver valuable comprehension for archaeological studies to fill knowledge gaps and further advance exploration of archaeological areas/features using RS along with DL approaches.

## 1. Introduction

Geospatial data derived from Remote Sensing (RS) standalone, e.g., Laser Scanning (LS) and photogrammetry, and combination approaches are being increasingly used for the digital preservation of archaeological information [1,2,3,4,5,6,7]. The effective application of these approaches is important, since there are hundreds of archaeological sites worldwide that have already been destroyed [8], and once an archaeological site is changed/distorted it is difficult to reconstruct it digitally without using appropriate and robust geospatial datasets. Some conservation studies, e.g., Moussa [1], Liang et al. [2], and Jaber and Abed [9], have used multi-RS data fusion to minimise the limitations (e.g., occlusion, Level Of Detail (LOD), precision) associated with standalone approaches and to generate relatively more detailed three-dimensional (3D) products.

Geospatial data have remarkable value in archaeological practice, as they are likely to contribute to the preservation of endangered sites for future generations [10,11]. These digital data can also be applied to discover new archaeological areas and detect previously unknown archaeological remains [12]. RS standalone and combination approaches in archaeology have a variety of applications, ranging from virtual reality consideration methods and 3D models to data storage, interpretation, and visualization; all possible without exposing sites to the prospect of demolition/excavation [13]. In parallel with the use of geospatial data, over the last several years, several archaeological studies have started applying Artificial Intelligence (AI) approaches, such as Machine Learning (ML) and Deep Learning (DL), to analyse RS datasets. These AI approaches are being used for the classification, identification, and segmentation of archaeological features.

The intention of this literature review is to review and critically discuss the findings of previous archaeological studies that have adopted RS (standalone and fusion/integration) approaches and DL techniques. This review highlights the importance of these approaches in detecting and preserving archaeological sites, and identifies a number of critical research gaps.

### Advanced Archaeological Techniques

Non-invasive techniques are applied in archaeological applications [14]. These techniques include RS standalone and combination approaches, and AI techniques are also used in archaeology. RS approaches are a further aspect of geophysical techniques. They are neither destructive nor invasive, and can accurately measure spatial data and attribute information (shape, size, areas) about AOIs.

**Figure 1:**
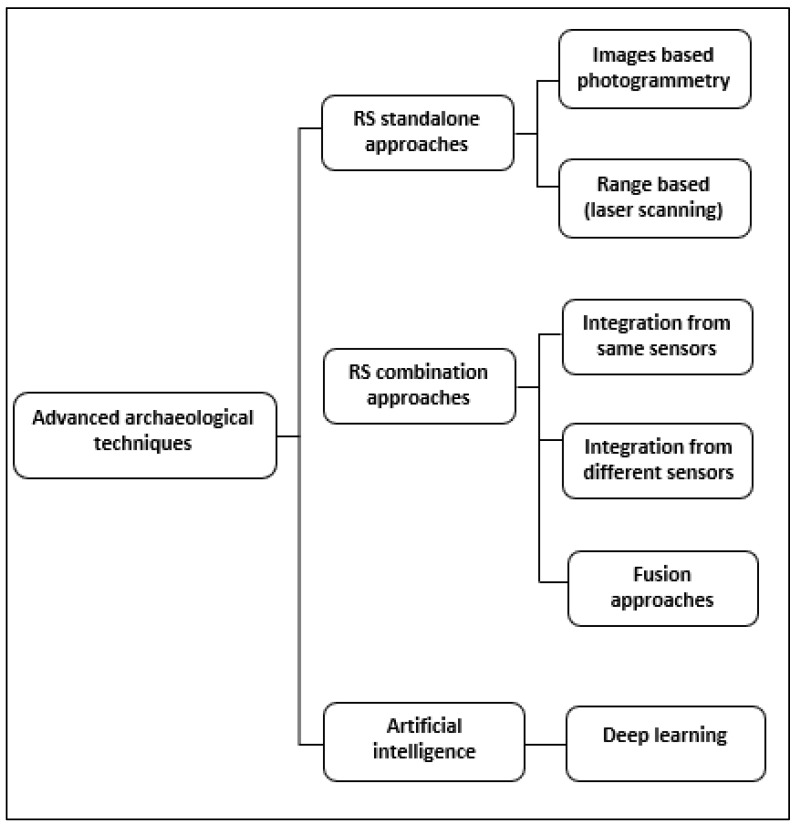
Advanced archaeological techniques. There is a research gap in assessing these approaches together to detect archaeological remains. These approaches are discussed in this review based on previous studies.

The previous review paper led by Adamopoulos and Rinaudo [15] discussed the applications of UAV-based remote sensing approaches reported in archaeology. In this paper, we aim to discuss the applications of RS approaches that are particularly based on laser scanning and photogrammetry in detecting and preserving archaeological remains. Many archaeological studies have applied RS standalone approaches (e.g., photogrammetry, Terrestrial Laser Scanning (TLS), and Light Detection and Ranging (LiDAR)) and combination approaches to reveal archaeological features and digitally preserve them. Object detection with DL is another advanced technique that has been adopted in archaeology in the last three years (since 2018) [16]. It semi-automatically detects objects based on raster images derived from RS approaches, as well as determines the likelihood of predicted features. These merits can be achieved through RS approaches without causing any damage/change to a site with respect to the original remains [17,18]. Previous studies are actively re-viewed and critically discussed in the following sections in order to identify research gaps. This review is divided into three sections: RS standalone approaches, including image-based modelling (photogrammetry) and range-based modelling, e.g., TLS and LiDAR; RS combination (integration and fusion) approaches; and object detection with DL (Figure 1). Previous studies have referred to combination, integration, fusion, and blending/merging terms as interchangeable terms. The research led by Kadhim et al. [19] referred to the fusion approach as a combination of 2D images with digital models (DSM/DTM) to create a fused model. In contrast, the term integration denotes a combination of raster images (2.5D image) derived from two different sources (LiDAR and photogrammetry).

**Figure 2:**
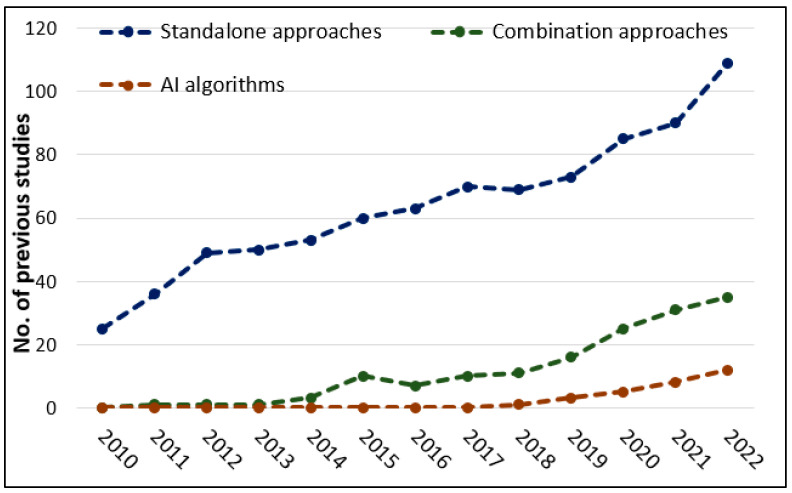
Literature count (2010–2022) from the database Scopus (http://www.scopus.com/) (accessed on 15 January 2023) for archaeological studies that applied Remote Sensing (RS) standalone and combination approaches (fusion/integration) and Artificial Intelligence (AI) in preserving and identifying archaeological features. The figure illustrates that there is a continuous and increasing trend of studies with the applications of LiDAR, photogrammetry, and AI techniques in archaeology—from 2010 to 2022.

Studies that adopted these approaches were collated from the database Scopus (http://www.scopus.com/) (accessed on 15 January 2023). Three filters were used to identify previous studies: For the standalone approaches, the key terms of “archaeology”, “hidden features”, “buried features”, “digital preservation”, “documentation”, “cultural heritage”, “3D modelling”, “3D reconstruction”, “GIS “, prospection”, “remote sensing”, “photogrammetry”, “aerial images”, “UAV” “Laser scanning”, “LiDAR”, “Terrestrial Laser Scanning” were used. The second filter was related to the RS combination approaches, and the key terms used were: “combination approaches”, “fusion”, “integration”, “merging”, “remote sensing”, “prospection”, “archaeology”, “cultural heritage”, “documentation”, “digital preservations”, “3D models”. Lastly, the third filter was for artificial intelligence, and the key terms used were “artificial intelligence”, “CNN”, “ANN”, “object detection”, “deep learning”, “training data”, “prospection”, “remote sensing”, “archaeology”. A number of scientific publications were obtained from the database Scopus (Figure 2). Results were categorized into three groups: (1) standalone, (2) combination, and (3) AI techniques in digital archaeology. Further relevant publications were identified from the citations/references of the studies identified from the database Scopus.

## 2. RS Standalone Approaches

As presented in Figure 2, many archaeological studies have applied RS standalone approaches, e.g., individually, TLS, LiDAR, and photogrammetry [7,13,18,20,21,22,23,24,25,26,27,28,29,30,31,32,33,34,35,36]. The main reasons for applying such approaches in archaeology are to assess the quality (accuracy and precision) of the delivered data, create 3D models, and discover hidden archaeological areas/features.

Photogrammetric models for archaeology had been rarely used prior to 2003, but since the emergence of the Structure from Motion-Multiview Stereo (SfM-MVS) method, it has been widely used for modelling historical areas [13,35,37]. LS has also become a popular technique to observe archaeological areas/constructions and create 3D models [38]. Early work by Hodge et al. [39] found that the high level of automated photogrammetric processing can provide an opportunity for efficiently creating visual data of AOI. However, the final products might not have adequate qualities for applications that require centimetre precision (such as monitoring deformation), which could be obtained from LS data. This finding also accords with the observations by Nuttens et al. [21], who carried out an accuracy assessment of TLS and photogrammetry by establishing Ground Control Points (GCPs) in a cultural site (Sint-Baafs Abbey in Belgium). They argued that these techniques should not only be applied to create 3D models, but also to determine the model accuracy of archaeological areas. Nuttens et al. [21] found that TLS errors were two times smaller than those obtained from photogrammetry. However, aerial photogrammetry is the most effective method for modelling the roof of archaeological constructions compared to other non-invasive methods, e.g., TLS and terrestrial photogrammetry, due to their inability to capture the top perspectives (rooftops) [1,9,26,40]. Accordingly, detailed digital 3D models could be, arguably, created by aerial photogrammetry.

The observations of TLS in archaeology were proposed by Nuttens et al. [21] and Hodge et al. [39], studies that established LS as an appropriate approach for modelling archaeological sites. The LS system has the capability to capture dense point clouds that can be processed to produce relatively highly accurate 3D models. Fassi et al. [22] disagreed and debated whether the accuracy and resolution of LS could be adversely affected by environmental conditions. For instance, wind, temperature, dust, raindrops, and fog are not ideal conditions for conducting fieldwork using LS [41,42]. A similar conclusion was reached by Grenzdörffer et al. [26], as they evaluated the limitations of the TLS and stated that the incident angles and the range (distance between laser beams and an object) relied on the reflection properties of the surface properties. The range measurement was also examined by Shanoer and Abed [29], who observed the Root Mean Square Errors (RMSE) of the TLS data for cultural heritage preservation. They found the RMSE of the minimum measurement range (3.5 m) in a Stonex X300 TLS device (www.stonex.it) (accessed on 27 November 2022) was 0.006 m, while the RMSE of a 7-m measurement range was 0.012 m. Therefore, the accuracy of the TLS modelling tends to be higher than those of photogrammetry, yet other factors (range measurements, incident angle, and surface properties) could adversely impact the quality of the modelling.

With regard to the data quality comparison between photogrammetry and LS, the work of Grenzdörffer et al. [26] determined the deviations between the LS and photogrammetry in modelling an ancient building (Cathedral of St. Nikolai in Germany) and found that the average deviations between the two observations were fluctuating from 0.02 m to 0.03 m. The outcome of the latter assessment means that the differences between these two techniques are not significant. The accuracy of the 3D models derived from photogrammetry was also examined by Hatzopoulos et al. [13], who employed the SfM photogrammetric method to reconstruct archaeological monuments/features. They yielded centimetre precision, relying on GCPs as well as camera exposure positions. Marín-Buzón et al. [36] also found that the SfM photogrammetry provided the most accurate data, compared to TLS data, for archaeological excavations. The main differences between LS and photogrammetry are summarised in Table 1. Shanoer and Abed [29] claimed that the TLS data must be interpreted and assessed based on the registration methods. They applied two fine registration algorithms—Levenberg-Marquardt Iterative Closest Point (LM-ICP) and Nearest Neighbour Iterative closest point (NN-ICP). The average registration errors were reported as 0.0026 m and 0.0039 m for the LM-ICP and NN-ICP, respectively.

The standalone RS approaches are not only applied to create detailed 3D models, but also to detect new archaeological areas/features. The use of these approaches for the detection of archaeological areas has been critically demonstrated in several archaeological studies [23,27,43,44,45,46,47]. More specifically, RS techniques including LiDAR and aerial photography can be adopted to both automatically and manually identify archaeological topographies [20,48,49]. LiDAR and Photogrammetry-derived digital models have been adopted in several archaeological projects to demonstrate how RS approaches can be used to identify, interpret, and assess the characteristics of archaeological sites [49,50,51,52]. For example, the DTMs generated from LiDAR data of Devil’s Furrow in the Czech Republic were used to identify terrain discontinuities, such as tracks and erosion furrows [23]. Bachagha et al. [43] illustrated the capability of DSM derived from LiDAR data along with 1-m resolution satellite imagery in identifying possible hidden ancient areas in Wadi El-Melah Valley in Gafsa, Tunisia. Based on spatial and pixel-based analysis methods, Bachagha et al. [43] discovered two possible Roman forts. These findings suggest that the combined application of RS datasets is a robust approach in archaeological prospection due to the detailed information that was obtained in terms of detection, localization, classification, and mapping of ancient features.

The raster images derived from DSMs/DTMs are being used towards the further successful detection of archaeological features [44,53]. Specifically, several Visual Analysis Techniques (VATs), e.g., hillshade, gradient, aspect, and Sky View Factor (SVF) derived from digital models, are applied to highlight topographic features of AOIs. VATs are adopted to identify topographic features and improve the understanding of archaeological areas. The gradient raster emphasises the altitude variations in AOIs, while aspect images show the directions of altitude variations [53]. In Bennett’s study [53], mounds and a potential new shell ring were detected through gradient and aspect images, as well as other VATS (e.g., hillshade). Cowley et al. [44] generated hillshade raster from LiDAR data (1 point/m^2^ point density). This raster successfully identified several archaeological features of AOI (Barwhill in the north of Gatehouse of the Fleet in Scotland). Examples of these detected remains are linear features that represent old water drainage and a Roman road. However, features could not be extracted from hillshade images, in some cases, the influence of the illumination in hillshade raster generates distortion and, in some cases, led to obscuring topographic features and hiding some archaeological remains [45]. Thompson and Prufer [27] support the claims of the previous observations, e.g., Bennett [53], as they observed that the LiDAR-derived hillshade, unlike the gradient raster, is not effective in recognising small structures. For this reason, they consider gradient raster images to be a robust alternative technique for detecting archaeological remains. Both Laser and photogrammetric data are being used to uncover archaeological information that might be unobtainable through the use of destructive excavation methods. The uses of standalone approaches (LS and Photogrammetry) in archaeology, experimental and analysis setup, their merits, and key findings from previous studies have been summarised in Table 2.

Therefore, RS data have been independently applied in many archaeological studies to evaluate how such approaches can be adopted to discover, interpret, and examine the physical characteristics of archaeological areas/objects [55,56,57,58,59]. Given that, there are strengths and weaknesses in both LS and photogrammetry. The range sensors are capable of creating 3D point clouds that are utilised later to produce fine geometric models [60]. In contrast, image sensors are more capable and appropriate for building 3D texture models of an object structure [60,61]. As such, combining the datasets is likely to create, to some degree, more complete 2.5D/3D models. Previous studies by Hatzopoulos et al. [13] and Dostal and Yamafune [60] argued that these methods could complement each other in generating relatively highly precise digital models of archaeological areas. More details about the combination approaches are discussed in Section 3.

## 3. RS Combination Approaches

The main purpose of combining multi-datasets is to address the limitations of the standalone approaches. Addressing these limitations is accomplished by combining multi-datasets derived from the same sensors and multi-sensors datasets. The development of both photogrammetry and LiDAR data in terms of quality and efficiency led some studies to recommend applying one technique over another to enhance the construction of digital models, as well as to improve the detection of archaeology [21,62]. An example of data integration from the same sensors is image-based modelling or range-based modelling [58,63], while an example of fusing/integrating different sensors is image-based modelling with range-based modelling [3,64].

Data integration from the same sensor has been applied in several archaeological studies, such as [57,58,63]. The concept of this approach is to combine two or more different raster layers derived from the same source (e.g., photogrammetry or LiDAR); this integration is based on the VATs. The intention of integrating multiple VATs is to address the limitations of single-raster images, generate newly enhanced datasets, and acquire clear topographical features of the AOI. The limitations of the standalone approaches are mainly associated with the illumination, distortions of the raster, and filtering [53,65]. Inomata et al. [57] suggested applying Red Relief Image Maps (RRIM) for object detection; RRIM is a VAT based on multi-layered topographic data; i.e., gradient and differential topographic data that are derived from the same sensor. It is a shade-free raster that signifies a fine feature of topographic data. This raster is normally used in archaeological studies, since it provides a clearer and less distorted view of topographic changes than the standalone VATs.

With regard to the RRIM, a relatively finer distinction of archaeological features (e.g., structures smaller than 50 cm) can be observed in the RRIM [57]. These results reflect those of Davis et al. [45], who found that the edges of archaeological features of Beaufort County, South Carolina, were emphasised in RRIMs. Kokalj and Somrak [58] and Kokalj et al. [66] corroborated the conclusions of the previous studies, as they suggested enhancing the existing individual VATs to improve archaeological prospection and avoid missing possible remains. Kokalj et al. [66] created open-access Relief Visualisation Tools (RVT) to integrate various raster images derived from various fine LiDAR data (e.g., 50 cm/pix and 25 cm/pix) in different archaeological areas to improve the visibility of the detected remains. Comparisons of various LiDAR-derived VATs (hillshade, gradient, and RRIM) and raster images from the RVT with 1-m/pix spatial resolution were implemented by Inomata et al. [57]. The results of the latter study showed that the edge of traces was relatively more emphasized in the RRIM than in other applied VATs.

In addition to the detection of archaeological remains based on integration approaches of VATs obtained from the same data source, combining multi datasets from different sensors is being applied to create relatively detailed 3D models for digital preservation. For instance, Papasaika et al. [67] applied the integration method to enhance accuracy, density, and reduced data voids. This enhancement is achieved by the combination of two different DSMs derived from different sources (LiDAR and IKONOS satellite imagery). The LiDAR data and IKONOS satellite imagery are different in terms of Ground Sampling Distance (GSD) and acquisition times. The weaknesses (e.g., voids, discontinuities) in the standalone data (e.g., LiDAR and satellite data) are often addressed in the fused/integrated data [67]. In accordance with the previous studies, other datasets were applied by Tapete et al. [68] to boost final outcomes. They combined synthetic aperture radar with TLS data to monitor the deformations of archaeological artefacts. Integrating these two techniques results in generating new datasets, consequently, enhancing data interpretation of segregations’ impact ancient monuments [68].

Recent archaeological studies, e.g., Liang et al. [6], Filzwieser et al. [69], and Luhmann et al. [40], found that combining multiple techniques is likely to boost the benefits of the acquired data to generate consistent and, to some extent, complete results. These approaches could complement each other to enhance 2.5D and 3D models of archaeological areas [13,60]. Therefore, the second approach discussed in this review is data combination from different sensors. This type of data combination is progressively becoming a vital factor in several RS applications, including archaeology [1,6,70]. Prior archaeological studies have noted the importance of combining multiple datasets derived from different sources (after individual processing) [1,3,5,40,64,70,71]. This approach includes the integration of 3D-to-3D dense clouds models [1,40,61,72], as well as 2D-to-2D raster images that were generated from photogrammetry and laser data [3,9,40] in order to generate integrated 3D models and digitally preserve ancient buildings/archaeological status.

The purposes for combining multiple datasets derived from different sensors have varied in individual previous studies. For instance, Forkuo and King [73] fused terrestrial photogrammetry with TLS data to generate realistic 3D models. The aim of their study was to develop a geometric relationship between the 2D digital images-based photogrammetry extracted from the LS 3D point clouds (the collinearity equations are often used for image-to-image registration to successfully merge multi-datasets that have different projections) [73]. To further support the idea of generating realistic models, an insight investigation on integrating photogrammetric and TLS data to create 3D models of a historical building (Villa Giovanelli) was carried out by Guarnieri et al. [61]. This study was based on highlighting the limitations of individual standalone approaches, as they used terrestrial photogrammetry to capture the basic detail, such as walls and facades, while more complex structures, e.g., turrets, statues, and the staircase, were determined by employing range-based modelling—Time Of Flight (TOF) TLS. The combination was executed by Guarnieri et al. [61] after establishing ten GCPs measured by total stations to geo-reference and merge both datasets towards achieving a realistic 3D integrated model. Thus, improving the visual quality and geometry of 2.5D/3D models is required to reduce or even eliminate occlusions in standalone data, generate relatively more detailed information, and digitally preserve archaeological sites.

The previous study led by Jaber and Abed [9] evaluated the effectiveness of the fusion approach in producing 3D modelling in both indoor and outdoor case studies; the statue of the Lady of Hatra (indoor case study) and Abbasid Mustansiriya School (outdoor case study) in Baghdad. The fusion was implemented by combining synthetic images derived from TLS point clouds and aerial images captured from a digital camera after identifying the limitations of individual sensors in archaeological preservation. An automatic co-registration scheme was executed, which involves the creation of synthetic images from the TLS to be combined with 2D images through the SfM method, and later applying simultaneous bundle block adjustment and Helmart transformation to eliminate discrepancies [64]. They found that fusing LS with digital images had significant benefits for digital preservation as it provided more detailed models by filling data occlusion and increasing the overall data density. Different RS datasets (e.g., digital images and LiDAR), in some cases, have varied data formats and projections. As a result, it is challenging to implement direct registration and identify common points to match both datasets [1,74]. The automatic registration of LS data and camera images was developed in Yanga et al. [74]; registration usually refers to LS aided by photogrammetry [5]. Moussa [1] stated that different types of methods can be used for registration, which can be categorised into manual registration and automatic registration; the latter method is highly preferable to minimise costs and time. The registration and the alignment processing with the Iterative Closest Point (ICP) algorithm can be applied with georeferencing processing. The registration errors between LiDAR and photogrammetric data (DSMs of Mount Cornello in Italy) did not exceed two meters. The resulting 3D model allowed for the identification and digitization of geologic features [75]. Thus, successful registration between two different datasets of the same AOI tends to achieve photorealistic 3D models.

As noted above, the combination approaches are mostly applied in archaeology to produce relatively more detailed 3D models than those obtained from the standalone approaches for digital preservation. Nonetheless, combination approaches, specifically LiDAR with photogrammetric data, are not commonly applied to improve the detection of archaeological remains. Specifically, limited archaeological studies have integrated/fused different datasets for object detection, such as [3,76,77,78,79]. From the archaeological perspective, the use of geophysics methods, e.g., Ground-penetrating radar (GPR) has facilitated the detection of unknown buried features [76]. The latter study by Deiana et al. [76] found that the integration of GPR with Electrical Resistivity Tomography (ERT) data into a single map can demonstrate an adequate method to interpret the geophysical anomalies and buried remains of Nora, in southern Sardinia. The detection of archaeological features using non-invasive techniques (geophysics and RS) should be implemented prior to the excavation process [76]. The purpose of applying such approaches is to examine the effectiveness of integrating/fusing multi-datasets obtained from different sources based on excavation evidence to accurately detect the presence of some hidden tombs. In this respect, Elfadaly et al. [77] are in agreement with Deiana et al. [76], as they also integrated the analysis of excavation and GPR with magnetic and aerial images of the Northern Nile Delta, Egypt to create an archaeological map that includes all the recorded and detected features/artifacts. Additionally, integrating radar data, topographic maps, and optical satellite imagery were applied to discover possible ancient settlement areas.

In terms of integrating LiDAR and photogrammetry, Holata et al. [3] created an integrated 2.5D model of the archaeological site (the deserted medieval settlement, Hound Tor, in south-west England) to digitally preserve the structure of the site, e.g., stone walls, the field remains (field enclosures, ridge, debris of constructions, and furrow). GCPs were established to georeference both datasets. Holata et al. [3] processed LiDAR point clouds to generate DSMs; unwanted points were removed (such as those captured by more than one flight line) in LAStools. They also generated a photogrammetric model of the AOI through the SfM-MVS method. The photogrammetric models can be converted to point clouds by applying the ‘Raster to point function’ in ArcMap [3]. The integrated DSM was achieved after georeferencing the point clouds obtained from LiDAR and photogrammetry. The key findings from previous studies are summarised in Table 3.

Therefore, the combination of various RS data generated from multiple sensors plays an important role in the interpretation and revealing of archaeological information. These approaches (e.g., fusing TLS with photogrammetric data) are mostly applied to improve the quality of 3D models by filling data gaps and increasing data density. Choosing appropriate combination approaches for a certain application relies on several factors, such as the complexity of AOIs, data availability, and the aim of the study. In spite of the merits acquired from the RS data in digital preservation, archaeological prospection, and detection, Guyot et al. [80] claimed that such detection based mainly on the VATs is time-consuming. Hence, DL algorithms could be used for automatic detection.

## 4. Object Detection with Deep Learning

With the considerable development of digital archaeology, several studies have focused on using DL Neural Networks (NN) to accelerate the object detection process and exceed output potential [30,81,82,83,84,85]. DL-NN, and in particular, ANNs and Convolutional Neural Networks (CNNs), are being used in pre-trained models and are being adopted in the automated mapping processing of archaeological areas. It has been found that applying DL algorithms in archaeology is likely to efficiently classify and identify ancient objects/features (saving time and cost) [86].

Several archaeological studies have found that DL-based LiDAR data has made remarkable contributions to digital archaeology. For instance, Somrak et al. [84] applied CNN with six different VATs (e.g., SVF, slope, hillshade, and positive openness) derived from LiDAR data to determine whether they can effectively classify archaeological structures (ancient Maya structures of Chactún area in Mexico). The classification was based on the Visual Geometry Group-19 (VGG-19) CNN and additional augmentation. VGG19 is an advanced CNN architecture with pre-trained layers, which is often applied to interpret the characterises of input data in terms of colour, form, and shape. Furthermore, [84] found that DL models using LiDAR-derived VATs without hillshade performed comparatively better than the models with hillshade. The overall classification (e.g., platforms, surrounding terrain, and constructions) of the LiDAR-derived VATs achieved 95% accuracy. Consistent with the previous study, i.e., Somrak et al. [84] and Trier et al. [30] found that DL had the potential for automatically mapping archaeological areas; they pre-trained 1.2 million images based on the LiDAR of the archaeological area in Arran, Scotland. The VAT used in the DL pipeline of the Trier et al. [30] study was the Local Relief Model (LRM) derived from LiDAR. The DL-NN was executed on the SLRM visualisations to classify three archaeological monuments (cairns, shieling huts, and roundhouses).

The latter study led by Guyot et al. [80] also demonstrated that DL algorithms can make significant contributions to archaeology, and they used them to reveal ancient structures of Tumulus du Moustoir in France. They also noted, however, that a large amount of input data is required to accurately train CNN models. With more training data, the more accurate the predictions that could be achieved [30]; this might clarify why most archaeological studies, as mentioned earlier, have applied LiDAR data on DL rather than aerial imagery [81]. LiDAR can be applied to capture raw data up to 300 m over AOIs, which means more coverage of an area can be obtained by airborne LiDAR than by UAV photogrammetry. Consequently, more datasets will be trained on the DL pipeline. LiDAR-derived VATs are being widely used for classification and segmentation due to their capability to generate a wealth of topographical information [87], specifically, in digital archaeology for large-scale mapping (e.g., 1:2500) [84].

Researchers have demonstrated several factors that could contribute to improving the accuracy of CNN models’ performance, such as the amount of training data, data augmentation, normalisation, and epochs [80]. The number of training data directly impacts the quality of DL functions; more training data are more likely to limit the overfitting of the CNN models. Trier et al. [30] argued that applying a large set of labelled data to pre-train the CNN models could potentially improve the identification of archaeological features. This argument was also supported by archaeological studies, such as Guyot et al. [80]; Küçükdemirci and Sarris [83], Somrak et al. [84], and Davis et al. [85], who recommended applying data augmentation in the DL pipeline to avoid adverse consequences (imprecise results) of the small datasets (e.g., less than 500). Guyot et al. [80] and Somrak et al. [84] corroborates the ideas of Trier et al. [30] and Maxwell et al. [87], who suggested applying data augmentation to enhance the performance of the CNN models and the ultimate results, even if a large number of datasets is used. Data augmentation has the capability to artificially enhance and transform (rotations/zooming/flips/scaling) the existing training data [87], but it does not generate new data. In other words, the purpose of using augmentation is to give a few other perspectives for individual datasets, consequently, limiting overfitting and making relatively better predictions [87]. Additionally, increasing the number of epochs, in some cases, leads to enhancing the validation accuracy, and thus, generating fit models [80,87].

Enhancing the validation accuracy and predictions of the DL CNN cannot only be achieved based on the augmentation number of epochs, but also on the normalisation function [30]. This function is normally used in the CNN models to scale and normalise outputs between 0 and 1 to rescale datasets without misshaping/deforming the variations in a range of values [88]. Hence, it contributes to making the CNN process relatively more effective and stable. Furthermore, Ioffe and Szegedy [89] found that Batch Normalisation (BN) is another powerful procedure to normalise the stimulations in the layers of DL CNN, Bjorck et al. [90] agreed and stated that the BN tends to speed up the CNN process and enhance training accuracy, since this technique can be performed on the input and intermediate layers [90,91]. The BN has been adopted in DL studies due to its capability to stabilise the CNN and simplify the optimisation process. Based on the arguments mentioned above, several aspects (training data, augmentation, normalisation, and epochs) should be considered toward enhancing the validation accuracy and predictions, as well as speeding up the DL-CNN performance. Previous archaeological studies are summarised in Table 4 using DL-CNN models. The review of the literature indicates that the application of DL CNN based on RS data, particularly photogrammetric data, in archaeology is still limited.

## 5. Conclusions and Future Work

The objective of this paper was to review and discuss the existing literature on the adoption of advanced techniques in digital archaeology, including RS standalone, combination approaches, and DL. This review provides an overview of how these approaches have been applied, with a specific focus on digital preservation and archaeological detection.

Despite the significant number of archaeological studies that have applied digital approaches, there are still knowledge gaps in investigating the application and accuracy of approaches, such as RS standalone, combination, and DL. Specifically, the integration of airborne LiDAR data with photogrammetric data is still not a commonly utilized method in archaeology, and there is also limited evidence of the use of combined approaches in detecting hidden remains. Furthermore, there has been a scarcity of research examining the limitations of standalone, integration, and fusion approaches when applied in combination to detect archaeological remains. This means that methods for applying standalone and combination approaches together in the same archaeological study have not yet been refined in an intensive and concentrated way. In addition, DL CNN models are still not commonly used in detecting archaeological remains. Thus, further assessment and articulation of various advanced approaches for the detection of archaeological features and digital preservation are critically needed.

To fill the knowledge gaps, our recent study (2023) led by Kadhim et al. [19] demonstrated a detailed workflow to investigate the potential of applying standalone, integration, and fusion approaches in detecting and recording archaeological remains of Cahokia’s Grand Plaza, Southwestern Illinois, based on aerial photogrammetry and LiDAR data. We argue that there is a high possibility that this investigation could make considerable further contributions to archaeological practice. In addition, various DL CNN models based on the RS datasets generated from both standalone and combination approaches should also be adopted in future archaeological studies. Improving the discovery of archaeological areas/remains using DL algorithms based on RS data is the most sophisticated and efficient way to identify possible new archaeological areas that have not been recorded in archaeological and cultural documents/archives.

## Figures and Tables

**Table 1:** The limitations of LS and photogrammetry.

Limitations	Photogrammetry	Laser Scanning
Accuracy	Centimetre accuracy (geotagged images).	Millimetre accuracy.
Modelling	Texturing and coloring are relatively better than LS.	Relatively better in penetrating and detecting features covered by dense vegetation.
Time	Data collection: depends on area coverage, number of exposures, overlap, speed. Processing: with advanced methods (e.g., the SfM), it might take less time than LS processing.	Data collection: scans thousands of points per second, but the time relies on area coverage, number of stations. Processing: it might take longer.
Cost (£)	>500 depending on drone and camera types.	Around 100,000
Weight (g)	~2000	Around 14,000

**Table 2:** A meta-analysis of some previous archaeological studies for Remote Sensing (RS) standalone approaches—Laser Scanning (LS) and image-based photogrammetry.

Study	Archaeological Site	RS Data	Finings/Conclusions
[32]	Chun Castle, UK	LiDAR and aerial photogrammetry	(I) Both LiDAR and photogrammetric data-derived VATs revealed archaeological features, such as huts/houses, linear features (possible paths), circular structures, and castle well. (II) In general, relatively less archaeological remains were detected by LiDAR data than those from photogrammetry. (III) The Red Relief Image Map (RRIM) of both data sources provided a comparatively higher level of detail compared to hillshade, aspect, and gradient raster images.
[31]	Cahokia Mounds, USA	LiDAR and aerial photogrammetry	(I) In some cases, photogrammetric data are appropriate alternatives to LiDAR data, specifically in areas with low vegetation coverage. (II) Aerial photogrammetry is faster and costs less than a LiDAR survey in observing archaeological areas. (III) Photogrammetry is relatively better in interpreting archaeological data due to its capability in generating true colour mosaics.
[45]	Beaufort County, South Carolina	LiDAR data	(I) Revealed 160 undetected mounds.
[44]	Barwhill in Scotland	LiDAR data	(I) Some archaeological remains (e.g., Roman roads and water drainage) were identified. (II) The influence of the illumination in LiDAR-derived hillshade (1-m spatial resolution) generates distorted raster, which led to the burying of some archaeological remains.
[29]	Lamassu and Sargon II the king of Assyria, Iraq	TLS and terrestrial photogrammetry	(I) Two TLS registration methods (LM-ICP and NN-ICP) were examined; the average errors were 0.004 m and 0.003 m for the NN-ICP and LM-ICP, correspondingly.
[13]	The Tholos of Delphi, Greece	Close range photogrammetry, TLS, GNSS	(I) A 3D map of ancient structures was created.
[28]	Palace Bridge, Russia	TLS data	(I) The draw spans of the bridge structure were reconstructed by creating 3D models. (II) TLS point clouds provided complete detail for modelling the bridge structure.
[27]	Uxbenká site core architecture, Toledo District, Belize	LiDAR data	(I) The Hillshade derived from LiDAR data (1-m spatial resolution), in some cases, provides a less robust method for revealing small structures if only LiDAR data is applied while gradient raster is relatively more effective in that case.
[26]	An ancient building ‘Cathedral St. Nikolai in Germany’	TLS, aerial photogrammetry, and total stations	(I) The standard deviations between models generated from the TLS and photogrammetry are not significant (between 0.03 m to 0.09 m) and variations in overlapping two models ranging between 0.02 m and 0.03 m, are determined through algorithms in CloudCompare.
[25]	Cotehele Quay, Cornwall in UK	LiDAR, TLS, and aerial images	(I) A realistic 3D model of Cotehele Quay was created. (II) Digital formats were translated from spatial data of coastal change to be available for general audiences. (III) Mixed-media films were designed to be used for climate and coastal change communications.
[24]	An old construction was built in 1874, Germany	Photogrammetry, TLS, total stations	(I) Photorealistic models were generated for digital visualization and reconstruction.
[23]	The southern part of Devil’s Furrow in the Czech Republic	LiDAR data	(I) Some archaeological features, such as tracks, pathways, and erosion furrows were detected and digitally preserved through various VATs, e.g., hillshade, gradient, and aspect images derived from LiDAR DTM.
[54]	An ancient building “Palazzo del Capitano”, Italy	TLS data	(I) Observing and monitoring ancient buildings.
[21]	A cultural site ‘Sint-Baafs Abbey in Belgium’	TLS, photogrammetry, and total stations	(I) 3D models of the AOI were created. (II) The horizontal and vertical accuracy of the TLS is two times higher than those generated from terrestrial photogrammetry.
[21]	Pinchango Alto, in the south of Lima, Peru	TLS and UAV imagery	(I) The standard deviation between models generated from TLS and photogrammetric data was 6 cm and the mean difference was less than 1 cm. These differences result from occlusions in both datasets.

**Table 3:** A meta-analysis of some studies that applied Remote Sensing (RS) combination approaches—Laser Scanning (LS) and image-based photogrammetry.

Study	Archaeological Site	Combination Approach	Findings/Conclusion
[64]	The Lady of Hatra (indoor statue), Al- Mustansiriya School, and Baghdad Qushla Tower (outdoor statues) in Iraq	Fusing TLS and digital aerial images	(I) 3D models of indoor and outdoor statues from TLS and photogrammetry. (II) Photogrammetry provides a comparatively denser, smoother, as well as more detailed model of the indoor statue than TLS. (III) The TLS model of the outdoor statues has a higher spatial resolution model than photogrammetric data. (IV) The Fusion of the two datasets has filled occlusions, produced more details by improving data density, and reduced the level of TLS data roughness.
[40]	Historic Churches in Georgia	Fusing TLS with terrestrial and aerial photogrammetry	(I) Both TLS and photogrammetry supply similar outcomes, but when both datasets are fused, a more complete 3D model is generated. (II) The aerial photogrammetry records the tower and roof of the construction that did not cover by terrestrial photos, nor TLS. (III) Applying the fusion approach through advanced software (e.g., RealityCapture) may save processing times and result in high-quality models.
[58]	Chactún area in Mexico, Celtic fields in Netherlands, and Julian Alps in Slovenia	Integrating VATs derived from LiDAR (same sensor)	(I) Combining visualization images can enhance the visibility and preserve the physical characteristics of the individual images. (II) The integrated outcome does not create artificial artifacts. (III) Applying a single visualization image is likely to miss valuable traces in the archaeological areas.
[3]	Hound Tor Deserted Medieval Village in south-west England	integrating LiDAR data with photogrammetry	(I) 3D enhanced, detailed, and precise model is produced from the integration approach. (II) Integration enhances the quality of the DSM/DTM created from low-resolution (1 pixel/m^2^) LiDAR data. (III) Various types of remains are digitised, such as farm fences, debris of buildings, ridges, and furrows in the study area. (IV) The main limitation of the results is that some parts of the study area have not been recorded by the SfM method due to the dense vegetation.
[75]	Mount Cornello, Southern Alps in Italy	Integrating aerial LiDAR and photogrammetric models	(I) Image point clouds achieved a relatively better 3D textured model than LiDAR point clouds. (II) Aerial LIDAR provides data of the flaws’ traces/geologic boundaries in areas covered with vegetation. (III) The integration of two models derived from airborne LiDAR and photogrammetric data results in a complete 3D model.
[1]	The temple of Heliopolis, Egypt and Hirsau Abbey in Germany	TLS and terrestrial photogrammetry	(I) The combination of synthetic images derived from TLS with digital images is an effective solution to overcome the limitations of the standalone data. (II) The combination approach resolved several issues including occlusions in TLS point clouds and providing 3D models with a higher level of detail.
[61]	Villa Giovanelli Colonna: a historical palace in Italy	Integrating TLS and terrestrial photogrammetric models	(I) The main structures of the palace (porch and façades) are modelled by image-based photogrammetry, while fine detail (staircase, turrets, and statues) are modelled by the TLS. (II) TLS point clouds need to be optimised to create adequate dense datasets. (III) Improper outcomes from image-based modelling were generated due to vegetation and shadows. (IV) A 3D complete and detailed model is generated from a combination of two RS data.

**Table 4:** Summarising some of the archaeological studies that applied Deep Learning—Convolutional Neural Networks (DL-CNN) models.

Study	Archaeological Site	Data Source	Findings/Conclusion
[30]	Arran in Scotland, UK	LiDAR	(I) Automatically mapping the archaeological area. (II) Three archaeological monuments (roundhouses, cairns, and shieling huts) were classified.
[83]	Demetrias site, Greece	GPR	(I) Anomalies identified.
[92]	Qanat systems of the Erbil, Kurdistan Region of Iraq	CORONA Satellite Imagery	(I) The qanat shafts were detected.
[84]	ancient Maya, Mexico	LiDAR	(I) Various types of ancient structures (building, terrain, aguada, and platform) were classified and distinguished. The overall accuracy exceeded 95%. (II) The performance of DL CNN models using VATs (without the hillshad raster) perform relatively better than models with the hillshade raster. (III) VATs derived from LiDAR are effective datasets for DL-based classification.
[80]	Tumulus du Moustoir site, France	LiDAR	(I) The DL-CNN accurately and semi-automatically identified and characterized archaeological anomalies.
[85]	Beaufort, Charleston, and George-town County in South Carolina, USA	LiDAR, SAR, multispectral	(I) The detection accuracy did not exceed 77%. (II) Over 100 shell rings were detected. (III) Preserving cultural deposits, as well as clarifying archaeological records.

## Data Availability

Not applicable.
